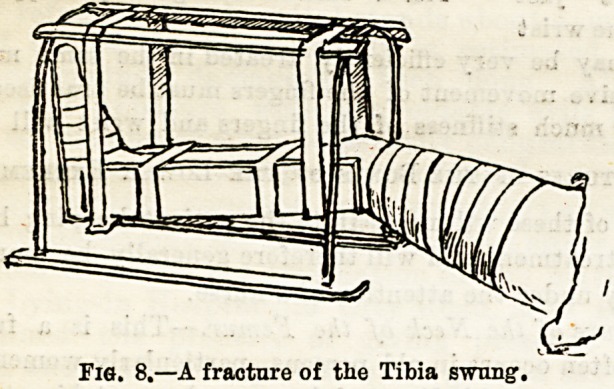# The Hospital Nursing Supplement

**Published:** 1895-03-30

**Authors:** 


### "The Hospital" Nursing Mirror.

BEING THE EXTRA NURSING SUPPLEMENT OF "THE HOSPITAL" NEWSPAPER.

[Contributions for this Supplement should be addressed to the Editor, THE HOSPITAL, 428, Strand, London, W.C., and should have the word "Nursing" plainly written in left-hand top corner of the envelope.]

### News from the Nursing World.

### UNDER ROYAL PATRONAGE.

THE Hospital and Home for Incurable Children, of which H.R.H. the Duke of Connaught is president, is also fortunate in its three Royal patronesses, Princess Christian, the Duchess of Connaught, and the Duchess of Teck. There was a large attendance at the annual meeting, held on the 23rd inst., at 2, Maida Vale, when Canon Duckworth took the chair, and, with other speakers, bore eloquent testimony to the valuable services rendered by Mr. Webb and the other honorary officers, and by the regular visitors. Most cordial appreciation was also shown of Miss Coleman's services, the esteemed matron, whose name was received with much applause. Various incidents were cited, illustrative of admirable administration, both in regard to finance and in such emergencies as unfortunately characterised the past winter, when half a score of the little patients contracted influenza, and two nurses and the cook were also ill, while there was the additional difficulty of frozen pipes. A more helpless party it would be hard to find, for the patients at 2, Maida Vale, are not only incurable, but in many cases more feeble than babies. An appeal was made for funds to construct a new day room for the children, the apartment in which the meeting was held having to serve as refectory, play, and class room in turn, an arrangement obviously inconvenient. Donations towards the sum required, only about £100, will be most gladly received and gratefully acknowledged.

### KING'S COLLEGE HOSPITAL.

THE development of the Nurse Training School at King's College Hospital has been so steady and gradual that it is difficult now to say at what precise date its completion was first acknowledged. Miss Monk, better known perhaps as "Sister Katherine," is one of the most enthusiastic of matrons, and her efforts in promoting the physical, moral, and intellectual welfare of her staff have long been recognised by the world outside as well as by the workers within the gates of King's College Hospital. In all her schemes she has been ably supported by Miss Peddie, the beloved "Sister Sibbald," whose death we report in another column. The classes held by Miss Peddie for the probationers were perhaps the special work in which she first signally distinguished herself. A long succession of pupils testify to the ever fresh interest she took in their instruction, never allowing her lessons to grow mechanical or monotonous. The excellent nurses' library, containing many valuable technical works as well as lighter literature, is another memento of personal interest as well as of the kind sympathy and substantial support which Sister Sibbald's wise plans received. Whilst mourning their own personal loss, her fellow-workers cannot but feel that death came to Sister Sibbald as she herself would have wished, in the midst of her work in her well-beloved hospital. It will be long ere her companions are reconciled to the absence of one thus called away in the prime of life and the fulness of power.

### HOSPITAL FOR CONSUMPTION.

HOWEVER deeply rooted may be our impressions regarding the sadness of consumption hospitals, they disappear rapidly during a visit to the fine buildings in the Fulham Road. In the bays which form day rooms for each long gallery, groups of cheerful patients take their meals, whilst flowers, piano, and books provide variety and employment. The absence of all draughts, and the even warmth maintained in the corridors and rooms alone suggest that these cheery folks are really patients under careful supervision and treatment. The superintendent, Miss Davidson, is enthusiastically interested in nursing, and she is proud of improved bedsteads recently imported for the patients, whilst rejoicing in the extended time off duty which she has brought into operation for her nurses. The wards, staircases, and waiting halls in the new block leave little to desire, and the beautifully ordered kitchen on the top floor is well nigh perfect, but the accommodation for the nurses does not fulfil modern requirements. We hope it will not be long ere the committee adds to its many other good arrangements that of providing a separate bed-room for each member of their nursing staff.

### DIVIDED DUTIES.

Two of the St. Olave's Guardians appear to be zealously discharging the duties entrusted to them by the ratepayers at the last election, as regards the supervision of the infirmary. Doubtless they were quite within their rights in paying a surprise visit to the wards at 11.30 p.m.; but whether the nurses could be considered as on duty with the patients whilst engaged in conversing in another room with the Guardians, one of whom was smoking, was justly questioned by the doctor. The matter was brought before the Guardians, one of whom finally announced that he should communicate the doctor's report to the Local Government Board. The difficulties of Poor Law nurses as well as of medical superintendents seem to be capable of infinite multiplication.

### ST. CATHERINE'S HOME.

THE second annual report of St. Catherine's Home, Bradford, is a peculiarly interesting one. It is very well got up, and printed by Mr. Gaskarth, of Bradford, and the rules and balance-sheet are placed before the reader in an admirable manner. The rent, rates, and taxes are a gift to the home, Mr. and Mrs. Bottomley having undertaken them for five years. Cases of cancer and advanced phthisis have the preference for admission, other incurable patients being also eligible. It is not possible to imagine any institution better deserving the title of home than this, for kindliness and courtesy pervade every detail of the management

The two-fold object of the home are described as the provision of medical care and nursing, and also the spiritual welfare of the patients, each having full liberty to be visited by a minister of her own choice. Results have abundantly justified our own conviction that in securing Miss Hadley Scott for the office of matron the committee had chosen a lady specially suited for the post. In the report now before us, the committee pleasantly testifies "to the good management of their most valued matron, Miss Hadley Scott, who, combining liberality with economy, and tact with firmness, conducts her sometimes difficult household to the admiration of all." It is easy to foresee a successful career for an institution in which kindly co-operation and substantial support are given by all.

### PROMPT MEASURES AT HINCKLEY.

The Isolation Hospital at Hinckley was reduced to a complete wreck by the gale on Sunday, the roof being carried some two fields away and the walls laid perfectly flat on the floors. The conduct of the nurses was admirable. Taking warning by the ominous shaking of the building they removed all the patients before the great crash came. About sixteen cases of diphtheria, chiefly children, were enveloped in blankets and borne out of the wards and laid in the shelter of a hedge until vehicles were requisitioned to take them to houses where they have found temporary refuge. The hospital stands about a mile and a half out of the town, and it is estimated that a thousand pounds damage has been done. Although fright and exposure were unavoidable evils for the patients, the nurses must be heartily congratulated on their prompt action and presence of mind.

### A MATRON'S OPINION OF PRESENTATIONS.

In acknowledging the handsome Indian silver tea-set presented to her on her resignation of the matronship of the Lying-in Hospital, in City Road, Miss Heanley condemned the practice of giving presents to the matron and staff of hospitals as too great a tax on the nurses and pupils, especially in lying-in hospitals, where a large sum is paid for a short training. The obvious truth of Miss Heanley's remarks must commend them to many of our readers. The kindly feeling which prompted this lovely parting gift was, however, most cordially appreciated and acknowledged in a little address to the nurses and pupils, in which Miss Heanley spoke on the present position of midwives, whilst looking forward to their holding a far better one in the future.

### THE "NURSES' JOURNAL."

We gather from a circular which has been issued by the secretary of the Royal British Nurses' Association that there is a general desire that the *Nurses' Journal* should be issued more frequently, and published at one penny for each number. It is proposed that the *Nurses' Journal* shall in future contain articles by well-known authors on subjects of general interest, in addition to the proceedings of the Royal British Nurses' Association and the report of its lectures. We understand that the large majority of the members of the Royal British Nurses' Association are favourable to this project. It is undoubtedly a move in the right direction, and we shall hope to soon have the pleasure of receiving each week a copy of the *Nurses' Journal* in its new form.

### CAGED ORPHANS.

One of this year's publications, issued by the Church Extension Society, contains very caustic remarks on persons who have made charges against the management of the Kilburn Orphanage, and although these accusations are not given in detail, it is implied that the treatment of the children has been called in question. No one who visits the orphans can doubt their healthy, well-nourished, and well-clothed condition, and this makes the persistent use of "cages" in the dormitories most incomprehensible. In The Hospital of December 1st, 1894, and February 9th, 1895, a sketch appeared of the so-called "comfortable little apartments," and the danger of these is obvious to all possessed of even the most elementary knowledge of the perils of fires, and of the helplessness of children. By removing the doors of each cubicle the evil would be reduced. When fastened they are dangerous, and when opened block the gangway to an extent which would be disastrous in an emergency. From the language in the magazine sent to us, it is evident that the good sisters resent the advice of "the ignorant but self-confident amateur,"—as is only natural. When, however, experts in institutional construction, and others possessed of large practical experience, remark on the dangers of the dormitory arrangements, the sisters will do well to reorganise them before an untoward accident makes action compulsory.

### INDIAN LADY DOCTORS.

With regard to the provision of medical aid for the women of India, the Countess of Dufferin has pointed out obvious advantages in its being supplied by persons born in India rather than by Englishwomen, although this decision disastrously limits the field of work of our fellow country-women. At first a Dufferin Fund chiefly granted scholarships to women promising to go out to India for five years after receiving a medical education at the London or Edinburgh School. In 1893 it was however decided that a special effort should be made to give similar advantages to women born in India, whether natives, Eurasians, or those of English parentage, who would be conversant with the languages and customs of the great Empire. It is believed by Lady Dufferin that economy would also result from the native hospitals being officered by Indian-born women. Those who have already been assisted by grants from the U. K. Branch of the Dufferin Fund are tried women who have, by their own desire, come to England to complete their studies and qualify for admission to the Medical Register. Unfortunately the ladies who have taste and ambition for the profession of medicine seem in India to be invariably without adequate means for pursuing their studies. Hence subscriptions to meet the demands on their resources are urgently demanded by the "National Society for Supplying Female Medical Aid to the Women of India." Subscriptions and donations will be gratefully acknowledged by the Marchioness of Dufferin and Ava, British Embassy, Paris; Miss Edith Heather Bigg, 11, Radnor Place, Hyde Park; also by Messrs. Coutts, 59, Strand, London.

### SHORT ITEMS.

We learn that Miss Patteson, the Matron of Lewisham Infirmary, resumed her duties there on the 20th inst.—Two thousand sailors attended the St. John Ambulance Classes held last year by Dr. Radford, and to twenty of these certificates have been awarded, which were presented last week by Lady Brassey at the Mission to Seamen Institute, East India Dock Road.—Miss Ravenhill, lecturer for the National Health Society, gave the last of an excellent course of lectures on "Domestic Economy" at Windsor on the 25th inst.—The Hampshire Nurses' Institute at Southampton, which supplies trained nurses for the sick in private families, has issued its twenty-seventh annual report.—The Duke and Duchess of Teck were present last week at a concert at the Star and Garter, given by the Wandering Minstrels, in aid of the funds of the Princess May Children's Ward at Richmond Hospital.—The Malmesbury Nursing Institute and Cottage Hospital report satisfactory progress in both branches.

### Elementary Anatomy and Surgery for Nurses.

By W. McADAM ECCLES, M.B., M.S., F.R.C.S., Lecturer to Nurses, West London Hospital, &c.

### X.—SPECIAL FRACTURES (*continued*).

### FRACTURES OF THE BONES OF THE UPPER EXTREMITY.

THERE are some points of practical importance in the treatment of fractures of the extremities concerning which it is advisable a nurse should have some knowledge.

*Fracture of the Clavicle.*—For a broken clavicle or collar-bone—a very frequent fracture—the most usual treatment adopted is that known as Sayre's method. This consists in

FIG. 1.—First piece of strapping applied.

FIG. 2.—Back view of second piece.

the application of two, and in some cases three, pieces of adhesive strapping in the following manner. The strips should be about three inches wide for an adult. The first piece should be long enough to encircle the arm and pass round the body. This piece is to be stitched loosely around the injured arm (which had better be carefully surrounded with wool in children) with the adhesive surface outermost. The strapping then having been carried round the trunk, adhering all the way, is sewn to the first part at the back. (Fig. 1.) The second piece has a length which will allow it to be fixed to the sound shoulder, carried down across the back, under the elbow of the injured side, a slit being made to take the olecranon process, and brought up to the sound shoulder again. (Figs. 2 and 3.) The third piece may be carried horizontally round the body, fixing the others in position. Lastly, a bandage is very conveniently applied over the strapping if the patient be restless. Care must be taken, especially in young patients, to see that no chafing result from the application of the strapping. Fractures of the clavicle will require to be kept at rest for about a month in an adult, three weeks in a child.

FIG. 3.—Front view of second piece.

*Fractures of the Humerus.* — A nurse may be required to prepare apparatus for the treatment of a fracture in the middle of the shaft of the bone. A rectangular splint, together with three short splints and suitable bandages will be required. (Fig. 4.) This situation is a frequent place for an ununited fracture to occur. It must be remembered that all splints applied to a fractured limb must be carefully padded, a duty which in hospital practice generally falls to the nurse. Especial attention must be paid to ensure that the padding at the ends of splints is full enough. In fractures about the elbow joints it is highly important to see that no pressure is brought to bear on the prominent bony points, and that passive movement is commenced before much stiffness of the articulation occurs. In a fracture of the olecranon process where the elbow joint has frequently to be kept in an almost fully extended position, it is advisable to prevent the arm from hanging down, for if it do so swelling of the hand is very likely to ensue.

*Fractures of the Radius and Ulna.*—Perhaps most usually two well-padded straight splints are applied in the more common forms of these fractures. Both splints must be a little wider than the forearm itself, and the length of the anterior splint must be such that it may not press into the bend of the elbow when this joint is flexed, and may allow free bending of the finger over the lower end; while the posterior splint should reach from just below the elbow to the knuckles. (Fig. 5.) A Colles's fracture, that is a fracture of the radius just above the wrist joint, may be very efficiently treated in the same manner, but passive movement of the fingers must be practised very early or much stiffness of the fingers and wrist will result.

FIG. 4.—Internal rectangular splint applied.

### FRACTURES OF THE BONES OF THE LOWER EXTREMITY.

Most of these will necessitate the patient keeping his bed during treatment and will therefore generally be more particularly under the attention of a nurse.

*Fracture of the Neck of the Femur.*—This is a fracture which often occurs in old persons, particularly women, from a small amount of indirect violence, such as catching the toes

FIG. 5.—Anterior splint for a Colles's fracture.

in the hearthrug. It is a serious accident, for by no means infrequently it leads to a fatal result from consequences which follow. Lying on the back has a tendency in all old people to cause congestion of the lungs, which is very insidious, and is apt to carry off such patients. Again, bedsores not infrequently arise. These patients require the utmost care and attention from the nurse.

*Fractures of the Shaft of the Femur.*—Of fractures this is *par excellence* the one that requires a properly appointed bed.

FIG. 6.—A Liston's long splint applied.

A firm mattress is absolutely essential, and the greatest care must be taken that no unevenness is allowed. A hospital nurse will be expected to prepare a Liston's long splint, reaching from the axilla to an inch or so below the sole of the foot. Strapping, stirrup, pulley, cord, and weight for extension, bandages both for the limb and body, two blocks to raise the foot of the bed will in addition almost certainly be required. (Fig. 6.)

*Fractures of the Patella.*—These are treated in very various ways, including the operation of wiring the fragments together. Much effusion of blood occurs into the knee

FIG. 7.—A Neville's back splint and a Salter's cradle.

joints, which the fracture almost necessarily involves. An ice-bag or evaporating lotions may be ordered.

*Fractures of the Bones of the Leg.*—Here there is often considerable danger of the fracture becoming compound if there is the least carelessness in manipulating the injured limb. In most hospitals the fracture is treated by means of

FIG. 8.—A fracture of the Tibia swung.

a Neville's back splint, and side splints, the whole forming the so-called box splint (Fig. 7.) When firmly fixed in this the limb is swung from a Salter's cradle (Fig. 8.) The nurse should see that the back splint has a sufficient hole in the position where the heel will rest, and to still further prevent pressure must provide some heel pads for the surgeon to use.

### Notes from Holland.

THE assistant-directress of the Binnen-Gasthuis, Amsterdam, Miss Cort van der Linden, is resigning the post which she has so ably held during eight years. Her training was received partly at the Hague, partly at the hospital at whose head she has since stood, and at St. Bartholomew's, where she worked as a probationer. She will be very much missed at the hospital, where her successor has not yet been appointed. The new asylum at Zuidlaren is to be opened in November of the present year. It will accommodate three hundred and fifty patients. The Idiot Asylum at Ermenloo has also been undergoing enlargement so as to contain about one hundred and twenty patients—seventy men and fifty women. The Society for Rendering First Aid in Cases of Accident is prospering at Rotterdam. Many applications to attend the courses of instruction organised by the society have been sent in; in fact, the number is largely in excess of those who can be received at present. It is proposed in the first instance to instruct the members of the police force and fire brigade, and men employed in large factories and in the docks.

### Death in Our Ranks.

IT is with the deepest regret we announce the sudden death on the 21st inst. at King's College Hospital of Miss Clara Sibbald Anderson Peddie, third daughter of Alexander Peddie, Esq., M.D., F.R.C.P., of Edinburgh, a gentleman who has long been revered and respected by a large circle of friends, and whose name has recently been before us as author of "The Life of Dr. John Brown," a work which has received considerable notice. After receiving her training in the Nightingale School at St. Thomas's Hospital, Miss Peddie, in July, 1884, became Sister of the Twining Ward in King's College Hospital, which post she held for four years, discharging the duties of her position with conspicuous ability and success. In 1888 Miss Peddie was promoted to the post of Home Sister and Lecturer to the Nursing School in King's College Hospital, which important office she held up to the day of her unlooked-for death. It was in this capacity that the great work of her life was done. In conjunction with Miss Monk (the well-known and devoted matron of King's College Hospital) Miss Peddie entirely organised and established the very excellent training school for nurses, for which the hospital is distinguished, and which will always remain a lasting memorial of her exceptional capacity and zealous devotion to the interests of nursing. Miss Peddie was held in the greatest respect and admiration by all the authorities of the hospital, and in her the institution has lost not only a valued worker but a substantial friend, her interest on several occasions having been the means of securing handsome donations to its funds. A memorial service, conducted by the Rev. Dr. Wace, Principal of King's College and chairman of the Committee of Management of the hospital, on Monday afternoon in the hospital chapel was attended by a large number of members of the Hospital Committee, the honorary and resident medical staff and the students, together with as many of the nursing staff as could be spared from the wards, and numerous private friends. The interment took place in Edinburgh on Tuesday morning, at Warriston Cemetery. Amongst many beautiful floral offerings from Scotch and English friends, was a beautiful bouquet of flowers with the following inscription :—

To her whose bright life's spring has now begun for her in heaven.—Clara Sibbald Peddie. We on earth send these spring flowers given us by her God and our God.—Florence Nightingale, March 21st, 1895, first day of spring.

And also a wreath of immortelles, bearing a card with the words :—

In deepest, saddest remembrance of her who has passed away from us so suddenly, but in joy and sympathy with her who, after so great a work in God's Hospital service that we feel she cannot be spared, has won the crown of work, in the arms of our Almighty Father.—Clara Sibbald Peddie.—Florence Nightingale sends this poor wreath, March 23rd, 1895.

Miss Peddie's memory will long be cherished by her fellow workers and the large band of nurses she assisted to train, whose respect and affection she had so completely gained. Her death, to her devoted friend, the matron, after ten years of unbroken intimacy and mutual regard, and to the hospital itself, is an irreparable loss.

### Where to Go.

UNIVERSITY EXTENSION SUMMER MEETING.—The seventh annual summer meeting of University and other students will be held in Oxford, from the 1st to 26th August. It will be divided into two parts, viz., August 1st to August 12th, and August 12th to August 26th. On August 12th excursions and a conversazione will be open to those holding tickets for either part. Applications for tickets, &c., should be sent to the secretary, Mr. J. A. R. Marriott, University Extension Office, Oxford.

### A Great Movement: The Nurses' Co-operation.

I.—INCUBATION.

IT is often said that women cannot co-operate, that they are so individualistic, so narrow, so selfish in their views, that they cannot see the use of working together. The assertion is no more true of one sex than of the other. There are two classes who never join a trades union—those who stand so high that they can command their own terms, and those who are so low that they are glad of any terms they can get. Unfortunately, there is a large number of women who belong to the latter class, and they tend to lower the price of their betters in all things in which quality is a matter of indifference. But when skill in work is an essential, women, as well as men, can co-operate in demanding its due recognition.

We purpose narrating the history of the largest co-operative movement that has yet taken place among women, a movement which has not only benefited the co-operators themselves but has had considerable influence in improving the condition of those whom we may term the non-unionists. The moral effect of the Nurses' Co-operation is as important as its practical results.

The history of many a nurse is pretty much as follows. She leaves the hospital meaning to undertake private work. The doctor under whom she has trained promises, perhaps, to "do all he can for her," and she takes a lodging in his neighbourhood. But no one doctor can keep a nurse fully employed, and you may be sure that there is more than one in his address book whom he can conscientiously recommend. At one time he could give her three cases simultaneously, at another he can provide her with none. Unless, indeed, he comes to her and says, "Oh, Nurse So-and-so, would you object to taking a case for me. The people aren't very well off, I fear they couldn't afford your regular terms, but if you would go for twelve and sixpence or fifteen shillings a week?" Well, she has been at home for six weeks, with rent going on, and food and fire to pay for. She takes the case, gives her intelligence, her training, her energy for the wages of a charwoman, and probably is grudged that. The people who pay inadequate wages always do grudge them. She may be thankful if her employers do not complain that she did not do the patient's washing and cooking, and help in the general house work. Next thing is that she is offered another case on the same terms. If she demurs she is reminded that she went to such-an-one for the same money, and her prices become permanently lowered.

Meanwhile other doctors, both in town and in the country, want nurses, and are sending for them. But where do they send? To a nursing institution. Not that they are unaware that there are many equally capable nurses who are independent, but they do not know where they live. Thus it naturally comes about that the nurse who has little or no work determines to join the staff of an institution. The pay is small, but it is sure, and she will have board and lodging when she is idle. But the institution nurse is rarely idle; the proprietor takes care of that. The staff is large enough to supply only the assured demand, and the nurse who once longed for work would now be glad of an idle day. It is all work and no play, and, what is worse, for next to no pay. The institution fees are high enough; but the nurse who earns £100 a year often receives only £20.

This is the story of—now many? The following letter, which appeared in THE HOSPITAL of February 2nd, 1889, records the experience of hundreds of others besides the writer:—

For the past two years I have been engaged in private nursing. How much I have wished I had stayed in the infirmary, comfortless though it was, would be hard to say, for then I could always think the poor patients were grateful to me for what I did for them, but now I feel nothing of the kind. The institution here belongs entirely (without a committee) to two nurses. The one aim is clearly shown in the treatment we receive by being sent off from one case to another (sometimes having been up the previous night) as being nought higher than money.

Another correspondent says:—

If, in addition to all the other good you have done for trained nurses, you could help them to get work for which they (not others) would be paid, we should all be grateful and glad to help you.

A scheme of help was already under consideration. In 1888 Miss Mary Belcher had written to THE HOSPITAL suggesting a combination of private nurses to protect their own interests. This led to a large correspondence, which showed how much some such association was needed. At the suggestion of the Editor of THE HOSPITAL a committee was formed to consider and formulate a scheme which would meet the circumstances. This committee consisted of Mr. Michelli, Mr. Ryan, Miss Belcher, Miss Napper, and Miss Honnor Morten. To most of our readers these names are well known. Mr. Michelli is the secretary of the Seamen's Hospital, and one of the most indefatigable of hospital workers. To his untiring devotion and energy not a little of the success of the Nurses' Co-operation is due. To the clear brain of Mr. Ryan, secretary of St. Mary's Hospital, Paddington, a well-merited tribute was paid when he gave evidence before the Lords' Committee on the London Hospitals. Miss Belcher was the originator of the scheme, and a representative private nurse; Miss Napper is matron of the Surrey Convalescent Home; and Miss Morten, who acted on the committee as Mr. Burdett's representative, is the author of the "Nurses' Dictionary" and of "How to Become a Nurse."

Thus every member of the committee was intimately acquainted with the needs of nurses and the conditions under which they work. Yet they did not at once fling out a scheme. They knew that to be successful it must be practical in every detail, and must, moreover, be supported by a sufficient number of doctors. It is in most cases the doctor who suggests getting a nurse, and he is to some extent held responsible for her proving acceptable or the reverse to the patient and the patient's friends. Therefore, in asking doctors of repute to support the Co-operation, it was necessary to give them a guarantee that its members should be such as they could recommend. Besides the qualifications of the nurses, there fell to be discussed the rules under which they should work—how much easier than the iron laws of an institution, how much straiter than the self-appointed regulations of the isolated nurse. Independence was to be respected, but some sort of homogeneity was necessary. So many points had to be considered that the committee had met and worked at Norfolk House for about a year before the Nurses' Co-operation was launched upon the world.

### The Metropolitan Hospital.

AN EVENING CONCERT.

AN excellent concert in aid of the Metropolitan Hospital was given on Wednesday, March 13th, by the Strolling Players' Amateur Orchestral Society in the Queen's Hall. The society had the valuable assistance of Madame Dotti, Mr. Ben Davies, and Mr. Santley, Mdlle. Henriette Murkens, who played the violin, and Master Redgrave Cripps, who presided at the piano. The programme was an excellent one, and the orchestra and various performers delighted a most appreciative audience. The Duke and Duchess of Teck were present, attended by a large party, and took their seats in a very prettily arranged portion of the grand circle. The decorations were tastefully carried out. During the evening the music of the "Metropolitan March" found customers amongst the company present, and was performed by the orchestra as the last piece on the programme. The Queen's Hall contained so large an audience that we trust the hospital has been materially benefited by the kind efforts of the organisers of the concert.

### Everybody's Opinion.

[Correspondence on all subjects is invited, but we cannot in any way be responsible for the opinions expressed by our correspondents. No communications can be entertained if the name and address of the correspondent is not given, or unless one side of the paper only be written on.]

### DISTRICT NURSING.

"A Constant Reader" writes: The articles on "District Nursing" in the "Nursing Mirror" of last year made me realise how much instruction is needed to fit even hospital nurses for the responsibilities of district nursing. It seems a pity that this is not better understood, for many people seem to believe that a knowledge of district nursing can be easily "picked up" by anyone with or without preliminary training. Now, if an intelligent woman wishes for proper theoretical and practical instruction in this branch of work where is she to get it? I shall be extremely grateful to any reader of The Hospital who can answer this question. There are, I know, various places where monthly or midwifery instruction can be had, but I find that the pupils do not often learn how to make the bed of the mother, nor are they taught to wash either her or the baby, and if these important details are ignored it is hardly likely that the true art of skilled nursing can be rightly appreciated. Your contributor in the articles already referred to showed so clearly that although district nursing is quite different to ward work, it is equally important, and should be performed equally well. Long experience of sickness in the homes of the poor has convinced me of the necessity for district nurses preparing themselves most thoroughly for their important duties. Many inquiries as to where such instruction can be gained have made me fear that Queen's nurses are almost the only women privileged to obtain systematic instruction.

[Nurses who have received full hospital training are admitted to the Central Training Home, Bloomsbury Square, for six months' instruction in district nursing when vacancies permit. At branch homes we believe the same arrangement is made for hospital-trained nurses.—Ed. *T. H.*]

### THE MALE SARAH GAMP.

A correspondent sends us the following authentic conversation between a military sister and her orderly: "Well, sister, I don't seem to be up to your ideas of a nurse, but I did good work amongst cholera, where no woman was, before ever I came into the Medical Staff Corps." "Tell me about it,' I said, still wondering why I found my orderly such a trying nurse whilst the medical officers all had such a high opinion of him. "I and another man were put in charge of three cases of cholera; we were put right away by ourselves in tents. The doctor prescribed for them, and left us to carry out his orders; so, as I was senior soldier, I arranged how the nursing was to be carried on. I said to the other orderly and the patients, 'There is a lot of fate about cholera; if you are to get better you will, and if you are not medicine and treatment isn't going to save you; so don't be frightened, and we shall get on very well.' I emptied the physic away and left them to nature, and what they fancied I saw they got." "And how did they get on?" I asked. "Oh, splendidly! The doctor, when he came to see them, said he never saw worse cases. He asked if I carried out his treatment carefully, and I said 'Yes,' meaning I carried it out of the tent, and he said no treatment seemed to influence them, they were so bad. Then two of them died and one recovered, and he said it was wonderful how I pulled that one through, and he recommended me for the way I looked after my patients; but you never seem to be satisfied when I am doing my best for them." "But a nurse must carry out her or his doctor's orders," I said. "Yes, of course, if he gives the right orders; but where would that cholera patient have been if I had done just as he said?"

### BURDETT'S OFFICIAL NURSING DIRECTORY.

"A. M." writes: Can you or any of your readers inform me what relation "C. Belasyse Myers," the writer of a letter in last week's *Nursing Record*, is to Mrs. Bedford Fenwick? Is the relationship that of sister, or brother-in-law, or neither?

### A CORNEY GRAIN MEMORIAL.

Mr. Arthur Lucas, chairman of the Children's Hospital, Great Ormond Street, writes from 27, Bruton Street, W.: Some friends of the late Mr. Corney Grain, remembering his love for children and how he delighted in entertaining them, have suggested that a fitting tribute to his memory would be the endowment of a cot bearing his name in the Hospital for Sick Children, Great Ormond Street. I have readily agreed to this suggestion, affording as it does, if carried into effect, an opportunity to many to evince their gratitude for the pleasure he gave by his kindly fun and healthy merriment. The endowment of a cot is £1,000, and subscriptions may be sent to the secretary, Mr. Adrian Hope, at the hospital.

### PRIVATE NURSING.

"A Private Nurse" writes: I would like to ask "L. B. R." what is meant by "keeping patient's room in order'? Simple dusting and arranging no true nurse could think of objecting to under almost any circumstances. Does "order" include the cleaning and black-leading of the grate? No one can reasonably expect a gentle, smooth touch from hands so employed. Of course, exception must be made of special cases where extreme quiet is necessary. How can "L. B. R." or anyone else expect a nurse to relieve them of anxiety by filling a post of responsibility and then ask her to take "her meals with the maids." If a nurse is fit to cope with mental and bodily illness and be a comfort in a house she should receive due consideration in her only free "sit down time," and have either solitary meals, or eat with people of higher education than the average maid servant, however worthy the latter may individually be. Personally I have always received the utmost courtesy, and trust that I shall always reciprocate it.

### Appointments.

Ulster Hospital for Children and Women, Belfast.—Miss Ethel Hoyle has been appointed Lady Superintendent of this hospital. She was trained at Leeds General Infirmary, and afterwards held the posts of head sister at Grimsby and District Hospital, sister of women's ward at Plymouth, and sister of children's ward at Portsmouth. We congratulate Miss Hoyle on her appointment, and wish her all success.

Throat and Ear Hospital, Brighton.—Miss May Poulton, who was trained at the Derbyshire Royal Infirmary, has been appointed Matron of this hospital. She was for two years charge nurse at the Lynn Hospital, then returned as sister to the Derbyshire Royal Infirmary, where she remained for eighteen months. We wish Miss Poulton every success in her new work.

Pearn Convalescent Hospital, Plymouth.—Miss C. Noel Thompson has been appointed Lady Superintendent of this convalescent hospital after five years' experience at Stockport Infirmary, where she held the post of matron. Miss Noel Thompson was previously matron of the Clayton Hospital, Wakefield, for two years. Her testimonials are excellent, and we wish her every success.

Bolton Workhouse Infirmary.—Miss Amy Hughes has been appointed Superintendent-Nurse of this infirmary. She was trained at St. Thomas's Hospital, and worked as nurse for the Metropolitan and National Nursing Association, afterwards becoming superintendent of the Branch Home at Chelsea. As superintendent of the Central Home, 23, Bloomsbury Square, a position which Miss Hughes is now resigning to take up infirmary work, she has proved an exceptionally good organiser, and her departure is regarded with genuine regret. The Guardians of Bolton are to be congratulated on their good fortune in securing Miss Hughes' valuable services.

### Ten Years' District Nursing.

It is with genuine regret that we chronicle the impending departure from London of Miss Amy Hughes. Since the completion of her training as a Nightingale Probationer Miss Hughes has devoted herself to district nursing, and under her supervision the work of the Central Training Home of the Metropolitan and National Nursing Association takes first rank in the civilised world. In every detail of duty Miss Hughes has shown by example and precept the boundless opportunities and the endless responsibilities of the trained district nurse. Thorough hospital training forms the invariable foundation on which is built up the qualified district nurse. No pupil of Miss Hughes has ever been permitted to assume that "it doesn't matter" if things are slurred over in the sick rooms of the poor. The bed of the district patient is as well-made as that of her sister in a hospital or infirmary ward. The room, however poor, is smartened into "nursing order," and the personal toilet of the sufferer is attended to with skill and tenderness which a millionaire might sigh for in vain. Miss Hughes will leave behind her a reputation which her successor may strive to copy but few women can hope to equal. Not only as a perfect nurse and practical housekeeper has she excelled, but also in the high tone and conscientiousness which are characteristic of all her work. Ten years of incessant application to the duties and superintendence of district nursing have shown to all who know her the conspicuous abilities possessed by Miss Hughes, and the work at Bolton Workhouse Infirmary cannot fail to prosper in her hands. It is probable that a new infirmary and a Nurses' Home will form portions of the improvements contemplated at no distant date at Bolton, and in securing such an able superintendent-nurse, the Guardians have given proof of their intentions to begin their reforms in the best possible way. Whilst regretting Miss Hughes' departure from the metropolis, we feel assured that her appointment under the Poor Law will be beneficial to a department where a vast field lies awaiting competent workers.

### Presentations.

On the 22nd inst., Miss C. Woolnough, District Nurse, St. Elizabeth's Home, Glasgow, was presented with a valuable gold watch by the women and girls of St. Mungo's Townhead. During the winter months, Nurse Woolnough gave a course of lectures on "Domestic Economy," and "Health and Sick Nursing," in connection with the night schools of that parish.

Miss Price, who has resigned her position as matron of Warneford Hospital, to be married, has been presented by the committee of management with a handsome silver salver and a Queen Anne tea and coffee service, in token of their appreciation of her seven years' excellent work in the institution.

### Minor Appointments.

Oriolet Cottage Hospital, Loughton, Essex.—Miss Gertrude Hick has been appointed Sister in Charge of this cottage hospital, which is chiefly for cancer cases. She was trained at the London Hospital, and takes many good wishes to her new work.

Bristol Royal Infirmary.—Miss Henrietta Hannath has been appointed Night Sister at the Bristol Royal Infirmary. Miss Hannath was trained at King's College Hospital, then worked at the National Hospital for the Paralysed and Epileptic, and for eighteen months held the post of home sister at the London Hospital. We wish her every success in her new work.

### Queen's Nurses at Aberdeen.

Last year 325 sick persons were nursed in their own homes by the Aberdeen District Association. This year 625 patients have received 14,894 visits. The staff is composed of a superintendent, four trained nurses, and one probationer. The latter is supplementing three years' hospital by special training in district nursing. The annual report, which is issued this month, makes the pleasing announcement that a large and convenient house has been purchased, and will be ready for occupation as a nurses' home about May. This will add greatly to the comfort of the nurses, and also afford facilities for the future increase of staff.

### Nursing in Paris.

A school for nurses of both sexes has been organised by the Municipal Council of Paris. Free courses are offered to those desirous of being initiated into the elements of nursing, and also advanced instruction is provided for those already possessed of some knowledge and anxious to attain to higher perfection. The classes are held twice a week in the amphitheatre of the "Hôpital International," and are open both to those desirous of obtaining diplomas of proficiency and to others. The lecturers already named are Dr. Aubean, Dr. L. Bonnet, Dr. Larriné, Dr. Bilhaut, Dr. Peisson, Dr. Fonquet, Dr. Paul Coruet, and M. Albin Rousselet.

### Notes and Queries.

### Queries.

(102) *Electrolysis.*—Is the operation for the removal of hairs from the face generally successful and can anyone give me some information about it?—*Sister R.*

(103) *Women's Hospitals.*—Could you tell me what hospitals there are in England for women only?—*Subscriber.*

(104) *Board.*—Can you tell me whether ladies are on the board of management of any English general hospitals?—*Urgent.*

(105) *Nurse.*—Please tell me if there is any harm in my wearing uniform and being called nurse, although I do not hold a certificate? I was a probationer in a Cottage Hospital and also took a district nurse's holiday work and am nurse and bath attendant in a hydropathic establishment.—*Hydro.*

(106) *Amputation.*—Has there ever been a practical test of artificial legs made by different makers, if so, whose was considered best? Judging them from appearance only is not sufficient.—*A Lady who Wears One.*

(107) *Epileptics.*—I shall be greatly obliged for the address of the society for giving employment to epileptics, and the name of the matron at the colony.—*Minister.*

(108) *Corsets.*—Is "The Khiva" a good substitute for ordinary corsets.—*District Nurse?*

(109) *Paralysed.*—Can you recommend a home for a girl?—*E.K.*

(110) *Imbecile.*—Please tell me what steps to take about placing an imbecile boy away from home.—*Anxious.*

### Answers.

(102) *Electrolysis (Sister R.).*—The operation for removal of hair by electrolysis is very often successful. The length of time required depends largely upon the number of hairs to be removed and the dose of pain the patient is willing to take at each sitting. The expense will, no doubt, vary, partly according to the time taken and partly according to the operator.

(103) *Women's Hospitals (Subscriber).*—You had better get "Burdett's Hospital Annual," published by the Scientific Press, 428, Strand, London, W.C.

(104) *Board (Urgent).*—There are ladies' committees which act under the board of management, but we do not know of any general hospital—except for women and children—where ladies are actually members of the board.

(105) *Nurse (Hydro).*—If you choose to wear uniform no one can prevent it. Does your employer prefer your being called nurse? Otherwise it would be far better for you to be styled attendant. A certificate does not make a nurse, it is merely a proof that she is fully trained. Sixteen months in a cottage hospital cannot be reckoned as sufficient training for a sick nurse, although it forms extremely useful experience.

(106) *Amputation (A Lady who Wears One).*—The surgeon who advises the purchase of an artificial limb, and decides on the date at which it should be taken into use, is the proper judge of the best kind for each case. It is a matter of great importance that the directions of the surgeon should be accurately transmitted to the maker of the limb, as he alone can judge of what is needed by his patient. Surely you do not imagine that people unlucky enough to be maimed, buy legs as lightly as walking sticks or bonnets?

(107) *Epileptics (Minister).*—Miss Delaney is the matron of the Epileptic Colony at Chalfont St. Peter, Bucks. Particulars can be obtained of the secretary, at the offices of the National Society for Employment of Epileptics, 12, Buckingham Street, Strand, London.

(108) *Corsets (District Nurse).*—We should advise your making a trial of the article in question. We cannot judge what would suit the patient you mention.

(109) (110).—You will find answers to both questions in the list of homes given in "Burdett's Hospital Annual," published at 428, Strand, London.

## Figures and Tables

**Fig. 1. f1:**
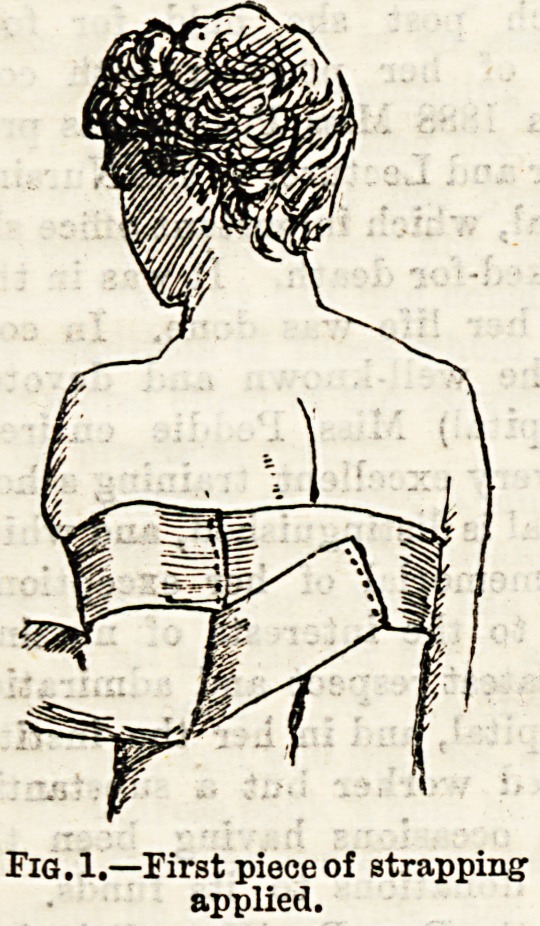


**Fig. 2. f2:**
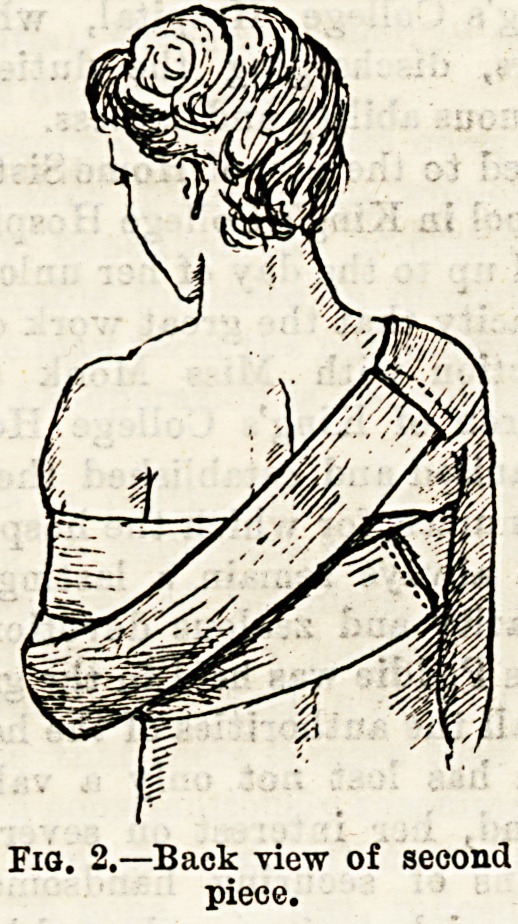


**Fig. 3. f3:**
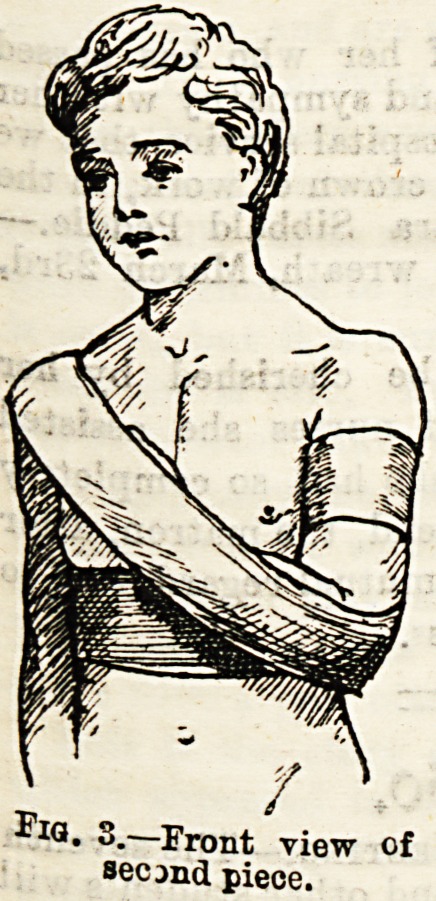


**Fig. 4. f4:**
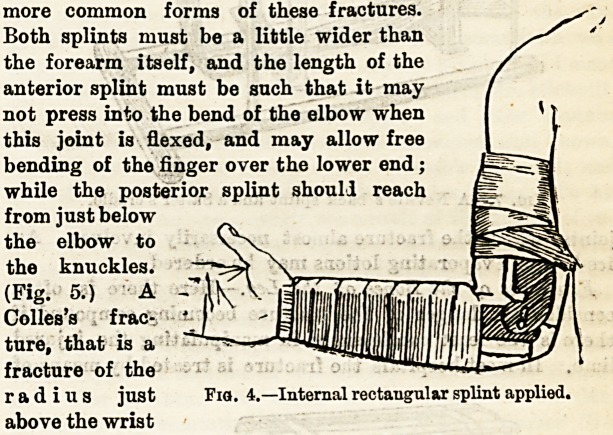


**Fig. 5. f5:**
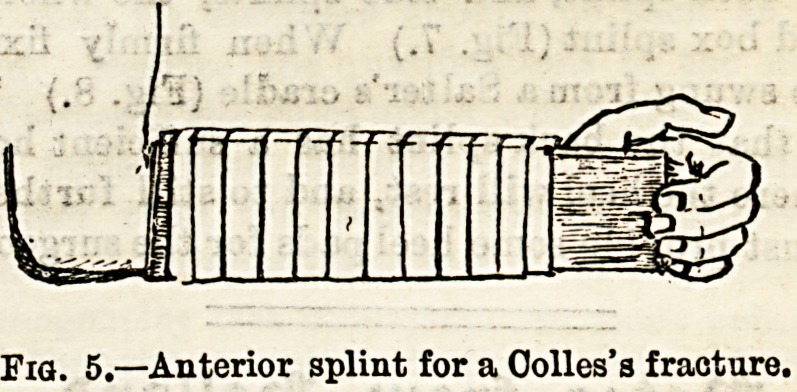


**Fig. 6. f6:**
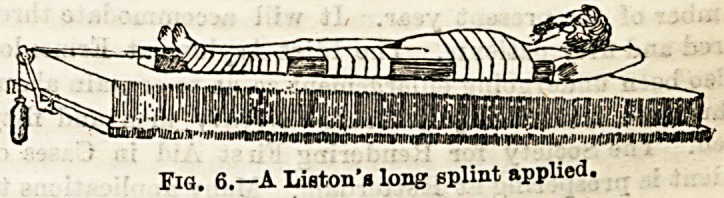


**Fig. 7. f7:**
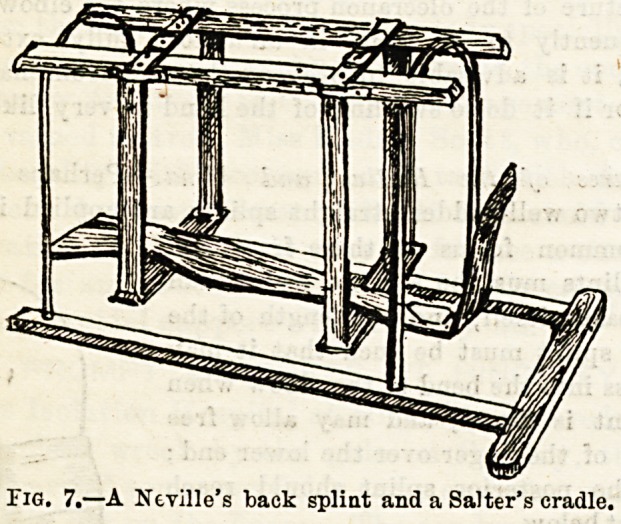


**Fig. 8. f8:**